# Trends in incidence and epidemiological characteristics of campylobacteriosis, Israel, 2013 to 2022

**DOI:** 10.2807/1560-7917.ES.2025.30.48.2500181

**Published:** 2025-12-04

**Authors:** Ravit Bassal, Shifra Ken-Dror, Merav Strauss, Miriam Parizade, Orli Sagi, Sharon Amit, Jacob Moran-Gilad, Orit Treygerman, Racheli Karyo, Iris Nasie, Noa Feldman, Maya Davidovich-Cohen, Assaf Rokney, Adi Sason, Lital Keinan-Boker, Dani Cohen

**Affiliations:** 1 Israel Center for Disease Control, Ministry of Health, Sheba Medical Center, Ramat-Gan, Israel; 2 Department of Epidemiology and Preventive Medicine, School of Public Health, Gray Faculty of Medical and Health Sciences, Tel Aviv University, Tel Aviv, Israel; 3 Clinical Microbiology Laboratory, Regional Laboratory Haifa and Western Galilee, Clalit Health Services, Nesher, Israel; 4 Microbiology Laboratory, Emek Medical Center, Afula, Israel; 5 Microbiology Mega Laboratory, Maccabi Health Services, Rehovot, Israel; 6 Clinical Microbiology Laboratory, Soroka University Medical Center and the Faculty of Health Sciences, Ben-Gurion University of the Negev, Beer-Sheva, Israel; 7 Clinical Microbiology Laboratory, Sheba Medical Center, Tel-Hashomer, Ramat Gan, Israel; 8 Clinical Microbiology Laboratory, The Department of Clinical Microbiology and Infectious Diseases, Hadassah Hebrew-University Medical Center, Jerusalem, Israel; 9 Central Laboratory, Meuhedet Health Services, Lod, Israel; 10 Central Laboratory, Clalit Health Services, Tel Aviv, Israel; 11 *Campylobacter* National Reference Center, Public Health Laboratories – Jerusalem, Public Health Services, Ministry of Health, Jerusalem, Israel; 12 School of Public Health, University of Haifa, Haifa, Israel

**Keywords:** Campylobacteriosis, Epidemiology, Incidence, Trend, Israel

## Abstract

**BACKGROUND:**

*Campylobacter* is one of the leading causes of gastrointestinal disease.

**AIM:**

We aimed to investigate trends in the incidence rate of campylobacteriosis in Israel.

**METHODS:**

We collected data on laboratory-confirmed cases of campylobacteriosis reported to the Israel Sentinel Laboratory-Based Surveillance Network (ISLBSN) in 2013–2022. Trends in the incidence rates of campylobacteriosis were evaluated using the Joinpoint software to calculate annual percent change (APC) and by time series analysis auto-regressive integrated moving average model.

**RESULTS:**

Between 2013 and 2022, 43,334 cases of campylobacteriosis were reported to the ISLBSN. The highest incidence rate of campylobacteriosis was observed in children aged 0-4 years (327.8/100,000) and overall, the incidence rate was higher among Jews and others (98.7/100,000) than among Arabs (85.9/100,000). However, the incidence rate among Arabs aged 0-4 years was higher (546.3/100,000) than among Jews and others (316.9/100,000). The incidence rate decreased significantly from 101.7 per 100,000 in 2013 to 79.4 per 100,000 in 2020 (APC = −2.7%) and then increased to 109.5 per 100,000 in 2022 (APC = 13.9%). We identified consistent peaks in incidence rate in April–May, specifically among Jews and others, with no corresponding increase among Arabs. Passover weeks were associated with a significantly higher risk of campylobacteriosis (incidence rate ratio (IRR) = 1.18; 95% CI: 1.12 to 1.23; p < 0.0001) compared with non-Passover weeks.

**CONCLUSION:**

Campylobacteriosis incidence rate in Israel is high, particularly among young children. Collaboration between veterinary and public health authorities and timely public awareness campaigns, especially before holidays, are essential to reduce zoonotic transmission and prevent future peaks.

**Key public health message**

**What did you want to address in this study and why?**

*Campylobacter* species are bacteria causing campylobacteriosis, a disease presenting with a range of symptoms including diarrhoea, vomiting and stomach pains. *Campylobacter* is the most common cause of food-borne infection in many countries. We aimed to estimate the trends and associated factors of *Campylobacter* infections in Israel.

**What have we learnt from this study?**

Campylobacteriosis is a common disease in Israel, with an estimated age-adjusted incidence rate of 83.0 per 100,000. Children under 5 years had the highest number of infections. The incidence rate increased in April–May, showing a seasonal rise during the Passover weeks among Jews and others (Jews, non-Arabic Christians and people with no defined religion), but not among Arabs (Muslims, Christian Arabs and Druze).

**What are the implications of your findings for public health?**

Our findings highlight the importance of targeted public health strategies that emphasise risk awareness, information sharing, and education on safe food handling and cooking practices. Strengthening food safety education, particularly in high-risk communities, may help reduce the burden of campylobacteriosis and prevent severe outcomes.

## Introduction

*Campylobacter* is recognised by World Health Organization (WHO) as one of the leading causes of gastrointestinal disease, particularly affecting young children, older adults and people with weakened immune systems [1]. Campylobacteriosis usually presents with diarrhoea, fever and abdominal cramps, but some patients are at an increased risk for post-infection complications, including Guillain–Barré syndrome, reactive arthritis and irritable bowel syndrome [2].

Birds are a natural reservoir for thermophilic *Campylobacter* species, as their intestinal tract offers an optimal biological niche for the survival and colonisation of *Campylobacter* [3]. *Campylobacter* is often transmitted to humans through the consumption of contaminated undercooked poultry meat and foods contaminated with bacteria through contact with raw meat or other sources due to cross-contamination [4]. Transmission can also occur via direct or indirect contact with animals infected with or carrying the bacteria and via direct contact with infected individuals [4]. Environmental factors, agricultural and food safety practices, integral to the One Health approach, influence the transmission of *Campylobacter.* Interventions such as improved biosecurity on farms, hygienic food handling and better monitoring throughout the food production chain can help reduce this risk and improve public health.

*Campylobacter* is less frequently identified as a cause of gastroenteritis outbreaks compared with some other enteric pathogens. This may be partly due to challenges in outbreak detection and under-reporting, given the lack of continuous surveillance in many countries. Among the outbreaks with an identified transmission source, most were food-borne, with chicken or chicken-containing dishes the most reported food vehicle [5].

*Campylobacter* spp. is highly prevalent in poultry in Israel. *Campylobacter* spp. was detected in 49 of 55 (89.1%, 95% confidence interval (CI): 80.9 to 97.3%) caecal samples and in 35 of 55 (63.6%, 95% CI: 50.9 to 76.4%) carcass samples from chicken slaughter batches collected between January 2015 and February 2016 in Israel [6].

Previously, we reported an increase in the incidence rate of campylobacteriosis from 65.7 per 100,000 population in 1999 to 101.7 per 100,000 in 2012 [7]. In the current study, we used the same sentinel laboratory-based surveillance network to update on the epidemiological characteristics and the trends of campylobacteriosis in Israel from 2013 to 2022.

## Methods

In Israel, laboratory testing of clinical specimens to diagnose campylobacteriosis is typically performed when requested by a physician for patients presenting with acute gastrointestinal symptoms, particularly diarrhoea, abdominal pain and fever, when bacterial enteritis is suspected.

### Study population

Data on laboratory-confirmed cases of campylobacteriosis from inpatients and outpatients were reported monthly to the Israel Sentinel Laboratory-Based Surveillance Network (ISLBSN), established in 1997 by the Israel Center for Disease Control (ICDC). The ISLBSN laboratories included were sentinel community and medical center laboratories throughout the country: northern Israel (Haemek Medical Center and Clalit Haifa Health Maintenance Organisation (HMO)), central Israel (Sheba Medical Center, Maccabi HMO laboratory with data from the formerly known Dan district and Clalit Petah-Tikva HMO), Jerusalem (Hadassah Medical Centers and Meuhedet HMO) and southern Israel (Soroka Medical Center), as presented in Supplementary Figure S1. No additional data were collected directly from the general practitioners. The population served by the ISLBSN laboratories was defined using data published annually from the Israeli Central Bureau of Statistics (ICBS) and the HMOs [8]. In 2018, the median year within the surveillance period, the ISLBSN laboratories covered 50.5% of the total Israeli population. Supplementary Table S1 presents the distributions of age, sex and population groups in the Israeli population and in the population served by the sentinel laboratories in 2015, to assess the demographic representativeness of the surveillance network.

### Case definitions

Between 2013 and 2019, we defined a case of campylobacteriosis as a person with *Campylobacter* spp. cultured from any clinical specimen type. From January 2020 onwards, PCR was used for detection of *Campylobacter* spp. genes directly from samples in the following laboratories: Haemek Medical Center, Clalit Haifa HMO, Soroka Medical Center, Clalit Petah-Tikva HMO, Maccabi Dan District HMO and Meuhedet HMO. Sheba Medical Center and Hadassah Medical Center continued to culture all samples.

A case was counted only once if more than one stool sample from the same person was positive for *Campylobacter* within a month.

### Detection of *Campylobacter* species

Culture for *Campylobacter* spp. was performed according to standard procedures by inoculating stool samples onto selective media and incubating them under microaerophilic conditions at 42°C for 48 h [9].

For PCR, a multiplex real-time PCR with the Seegene Allplex GI-Bacteria (I) kit (Seegene, Seoul, South Korea) was used. PCR results were not routinely confirmed by a secondary method. The use of the PCR assay led to an approximate 36% improved sensitivity vs culture without a noticeable decrease in the clinical specificity of *Campylobacter* spp. detection [10].

Of the cases reported to the ISLBSN, a random monthly sample of 15 isolates was sent to the *Campylobacter* National Reference Center for identification of the *Campylobacter* species (*C. jejuni, C. coli* and others) using an in-house multiplex PCR as previously described [11]. Isolates were selected irrespective of age or sex and were not stratified by demographic characteristics.

### Demographic data

For each case, the following data were collected, based on the data reported by the sentinel laboratory: age (0–4, 5–9, 10–14, 15–19, 20–24, 25–34, 35–44, 45–54, 55–64 and ≥ 65 years), sex (male and female), birth country (Israel and other), population group (Jews and others: Jews, non-Arabic Christians and people with no defined religion; Arabs: Muslims, Christian Arabs and Druze), district of residence (Jerusalem, North, Haifa, Central, Tel-Aviv, South, and Judea and Samaria) and socioeconomic rank. The socioeconomic rank was allocated using the socioeconomic residential classification published by the ICBS in 2015. This classification is based on 14 variables including demographic characteristics, education level, employment and lifestyle. A score ranges from 1 (lowest) to 10 (highest), reflecting the relative socioeconomic status by residential areas [12]. Information on the specimen type (e.g. stool and blood) was also collected.

### Statistical analysis

Annual and monthly estimated age-adjusted incidence rates were calculated per 100,000 population using direct standardisation, based on the age distribution of the Israeli population in 2015. Incidence rates were calculated per 100,000 population and stratified by age, sex and population group. Incidence rate ratio (IRR) and 95% CI were calculated to compare incidence rates of campylobacteriosis across age, sex and population group. We calculated IRRs and 95% CI using standard log-transformation methods, assuming a Poisson distribution of cases. Data analysis was carried out using SAS version 7.12 (7.100.2.3350) (SAS Institute Inc., Cary, the United States (US)). Secular trends in estimated age-adjusted incidence rates were evaluated using the Joinpoint Regression Programme version 4.9.0.0 [13]. The analysis was conducted on yearly age-adjusted incidence rates, and the model allowed up to one joinpoint. The annual percent change (APC) and corresponding 95% CI were calculated to describe the magnitude and direction of trends between joinpoints in the modelled estimated age-adjusted incidence rates. Weekly incidence rates were analysed using a time series model based on the autoregressive integrated moving average (ARIMA) approach to assess temporal patterns and evaluate model fit. Model adequacy was tested using the Ljung-Box test, which evaluates whether residuals are independently distributed (i.e. no significant autocorrelation). The analysis was generated using IBM SPSS software version 29 (IBM, Armonk, US). To assess the effect of Passover on campylobacteriosis incidence rates, we conducted a Poisson regression analysis using SAS, selecting this period based on the temporal clustering of morbidity peaks. The model included week number and a binary variable (0 = non-Passover week, 1 = Passover week) as explanatory variables. The model used a log link function, and results are presented as IRRs with corresponding 95% CI. The analysis was structured as an interrupted time series to compare incidence rates before and after Passover periods. A two-sided p of < 0.05 was considered statistically significant for all statistical tests performed.

## Results

### Descriptive analysis

Between 2013 and 2022, 43,334 cases of campylobacteriosis were reported to the ISLBSN. Of these cases, 9,991 (23.1%) were reported by Soroka Medical Center, 9,873 (22.8%) by Clalit Haifa HMO and 8,642 (19.9%) by Meuhedet HMO. Data completeness was generally high, with the highest proportion of missing values observed for population group (3.9%). Of the 43,152 cases with information on age available, 17,897 (41.5%) were aged 0–4 years, 7,221 (16.7%) were aged 5–14 years, 4,050 (9.4%) were aged ≥ 65 years and data on age were missing for 182 cases. Of the 43,239 cases with information on sex, 23,796 (55.0%) were males, 19,443 (45.0%) were females and data on sex were missing for 95 cases. Of the 42,703 (98.5%) cases with data on country of birth available, 37,495 (87.8%) were born in Israel. Of the 41,671 cases with information on population group, 33,993 (81.6%) were Jews and others and 7,678 (18.4%) were Arabs. Geographically, 10,032 (23.3%) of 42,989 cases with available data resided in the southern district, 7,619 (17.7%) in the Jerusalem district and 7,200 (16.7%) in the northern district. The median socioeconomic rank was 5.0 (interquartile range (IQR): 3–7). Specimens were predominantly stools (99.6%), only 0.4% were blood samples. Supplementary Table S2 depicts the demographic characteristics of the cases.

### Incidence rate of campylobacteriosis

**Figure 1:**
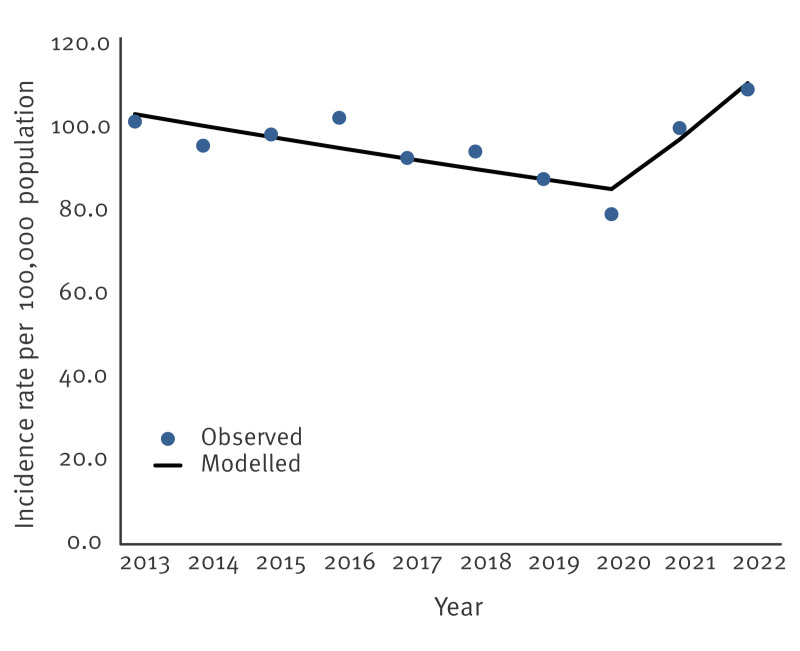
Observed and modelled estimated age-adjusted incidence rates of campylobacteriosis, Israel, 2013–2022 (n = 43,334 cases)

The overall incidence rate was 84.8 per 100,000 population and the estimated age-adjusted incidence rate was 83.0 per 100,000. Figure 1 displays the observed and modelled estimated yearly age-adjusted incidence rates of campylobacteriosis between 2013 and 2022. In 2013, the estimated age-adjusted incidence rate was 101.7 per 100,000, and it decreased significantly to 79.4 per 100,000 in 2020 (APC = −2.7%; 95% CI: −4.5 to −0.8%; p = 0.014), coinciding with the first year of the COVID-19 pandemic, and increased to 109.5 per 100,000 in 2022 (APC = 13.9%; 95% CI: −1.2 to 31.4%; p = 0.065) (Table 1).

**Table 1:** Joinpoint regression analysis of incidence rates of campylobacteriosis, Israel, 2013–2022 (n = 43,334 cases)

Variable	Year	Joinpoint year	APC (%)	95% CI (%)	p value	TI
Age						
Age-adjusted	2013–2020	2020	−2.7	−4.5 to −0.8	0.014	SD
	2020–2022		13.9	−1.2 to 31.4	0.065	NSI
Age group (years)						
0–4	2013–2022	NJ	−2.5	−4.7 to −0.2	0.037	SD
5–14	2013–2020	2020	−3.8	−7.4 to 0.0	0.050	NSD
	2020–2022		20.9	−9.2 to 61.1	0.149	NSI
15–19	2013–2020	2020	−0.9	−4.8 to 3.0	0.565	NSD
	2020–2022		17.1	−12.7 to 57.1	0.226	NSI
20–24	2013–2020	2020	−2.2	−4.8 to 0.5	0.086	NSD
	2020–2022		17.8	−3.8 to 44.2	0.093	NSI
25–34	2013–2020	2020	−1.6	−4.0 to 0.7	0.135	NSD
	2020–2022		19.1	−0.5 to 42.5	0.055	NSI
35–44	2013–2022	NJ	2.3	−0.8 to 5.4	0.129	NSI
45–54	2013–2022	NJ	4.0	0.2 to 7.9	0.043	SI
55–64	2013–2022	NJ	1.2	−1.3 to 3.7	0.296	NSI
≥ 65	2013–2020	2020	1.6	−0.8 to 4.2	0.153	NSI
	2020–2022		23.7	2.9 to 48.7	0.031	SI
Sex						
Male	2013–2020	2020	−2.7	−4.9 to −0.3	0.032	SD
	2020–2022		17.0	−1.9 to 39.6	0.071	NSI
Female	2013–2020	2020	−2.0	−3.6 to −0.5	0.021	SD
	2020–2022		13.3	0.6 to 27.7	0.043	SI
Population group						
Jews and others	2013–2020	2020	−3.5	−5.5 to −1.4	0.007	SD
	2020–2022		16.4	−0.4 to 36.1	0.055	NSI
Arabs	2013–2022	NJ	0.7	−2.1 to 3.7	0.572	NSI

APC: annual percent change; CI: confidence Interval; NJ: no joinpoint; NSD: non-significant decrease; NSI: non-significant increase; SD: significant decrease; SI: significant increase; TI: trend Interpretation.

Joinpoint regression results showed a significant decrease in age-adjusted campylobacteriosis incidence rates between 2013 and 2020 among males (APC = –2.7%; 95% CI: –4.9 to –0.3; p = 0.032) and females (APC = –2.0%; 95% CI: –3.6 to –0.5; p = 0.021) (Table 1). A significant decrease between 2013 and 2020 was also observed among Jews and others (APC = –3.5%; 95% CI: –5.5 to –1.4; p = 0.007) (Table 1).

### Incidence rate by age, sex and population group

#### Age

The highest incidence rate of campylobacteriosis was observed in children aged 0–4 years, with a rate of 327.8 per 100,000 population (95% CI: 323.0 to 332.6/100,000). This was followed by children and adolescents aged 5–14 years (74.2/100,000; 95% CI: 72.5 to 76.0/100,000) and adolescents aged 15–19 years, with rates of 68.2 per 100,000 (95% CI: 65.7 to 70.8/100,000) and young adults aged 20–24 years with 72.4 per 100,000 (95% CI: 69.7 to 75.1/100,000). Among individuals aged 25–34 years, the incidence rate was 53.2 per 100,000 (95% CI: 51.5 to 55.0/100,000), decreasing to 27.7 per 100,000 (95% CI: 26.4 to 29.0/100,000) among those aged 35–44 years and 27.1 per 100,000 (95% CI: 25.7 to 28.6/100,000) in the 45–54 years age group. Among adults aged 55–64 years, the rate was 38.0 per 100,000 (95% CI: 36.2 to 39.9/100,000), while an increase was observed in older adults aged ≥ 65 years, with a rate of 75.4 per 100,000 (95% CI: 73.1 to 77.7/100,000). While overall incidence rates varied by age, Supplementary Figure S2 illustrates general seasonal patterns over time for each age group, though detailed fluctuations are less pronounced.

#### Sex

There were more campylobacteriosis cases in males (107.4/100,000; 95% CI: 106.0 to 108.7/100,000) than in females (86.2/100,000; 95% CI: 85.0 to 87.4/100,000), as presented in Supplementary Figure S3, with significantly higher incidence rates and IRR among males aged 0–4 years (IRR = 1.28; 95% CI = 1.24 to 1.32), 5–14 years (IRR = 1.58; 95% CI: 1.50 to 1.65) and 15–19 years (IRR = 1.97; 95% CI: 1.82 to 2.13), compared with females (Table 2). In contrast, there were more cases in females than in males in the age groups 25–34, 35–44 years and 55–64 years (Table 2).

**Table 2:** Incidence rates of campylobacteriosis by age, sex and population group, Israel, 2013–2022 (n = 43,334 cases)^a^

Age group (years)	Sex				Population group			
	Incidence rate per 100,000 population		Incidence rate ratio		Incidence rate per 100,000 population		Incidence rate ratio	
	Males	Females	Males vs females	95% CI	Jews and others	Arabs	Jews and others vs Arabs	95% CI
0–4	427.6	333.7	1.28	1.24 to 1.32	316.9	546.3	0.58	0.56 to 0.60
5–14	104.3	66.1	1.58	1.50 to 1.65	102.3	30.3	3.38	3.11 to 3.67
15–19	102.2	51.9	1.97	1.82 to 2.13	100.4	16.1	6.22	5.30 to 7.30
20–24	82.6	84.3	0.98	0.91 to 1.06	104.8	14.7	7.15	5.97 to 8.55
25–34	56.3	68.5	0.82	0.77 to 0.88	74.0	17.0	4.36	3.80 to 5.00
35–44	29.9	33.8	0.88	0.80 to 0.97	35.2	15.4	2.29	1.94 to 2.70
45–54	31.7	30.4	1.04	0.94 to 1.16	33.3	19.1	1.74	1.48 to 2.06
55–64	41.5	47.2	0.88	0.80 to 0.97	47.2	25.8	1.83	1.53 to 2.19
≥ 65	84.1	82.4	1.02	0.96 to 1.09	86.3	47.5	1.82	1.57 to 2.10
Overall	107.4	86.2	1.24	1.22 to 1.27	98.7	85.9	1.14	1.12 to 1.18

CI: confidence interval.

^a^ Information on age was available for 43,152, information on population group for 41,671 cases.

#### Population group

Overall, the incidence rate of campylobacteriosis was higher among Jews and others (98.7/100,000; 95% CI: 97.6 to 99.8/100,000) than among Arabs (85.9/100,000; 95% CI: 84.0 to 87.9/100,000). However, the incidence rate among Arabs aged 0-4 years was (546.3/100,000; 95% CI: 532.5 to 560.4/100,000), higher than in Jews and others (316.9/100,000; 95% CI: 311.0 to 322.8/100,000). Notably, in the first year of life, the mean incidence rate of campylobacteriosis among Arabs was substantially higher (1,581.6/100,000; 95% CI: 1,529.8 to 1,635.1) than among Jews and others (283.5/100,000; 95% CI: 271.5 to 295.9), as depicted in Supplementary Figure S4.

### Seasonal effect

**Figure 2:**
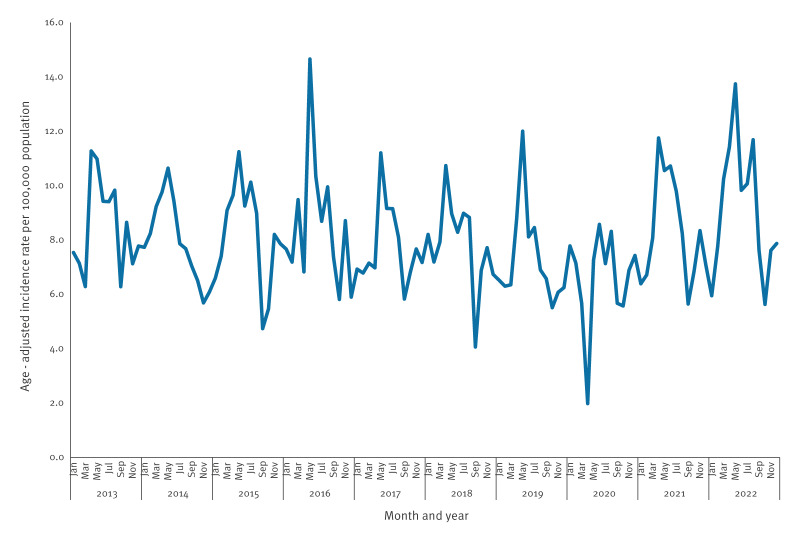
Estimated age-adjusted incidence rate of campylobacteriosis, by month, Israel, 2013–2022 (n = 43,334 cases)

The monthly incidence rate of campylobacteriosis showed a consistent peak every year during the spring months April–May, with elevated rates occasionally extending in the summer months June–July but declining from August through October (Figure 2).

**Figure 3:**
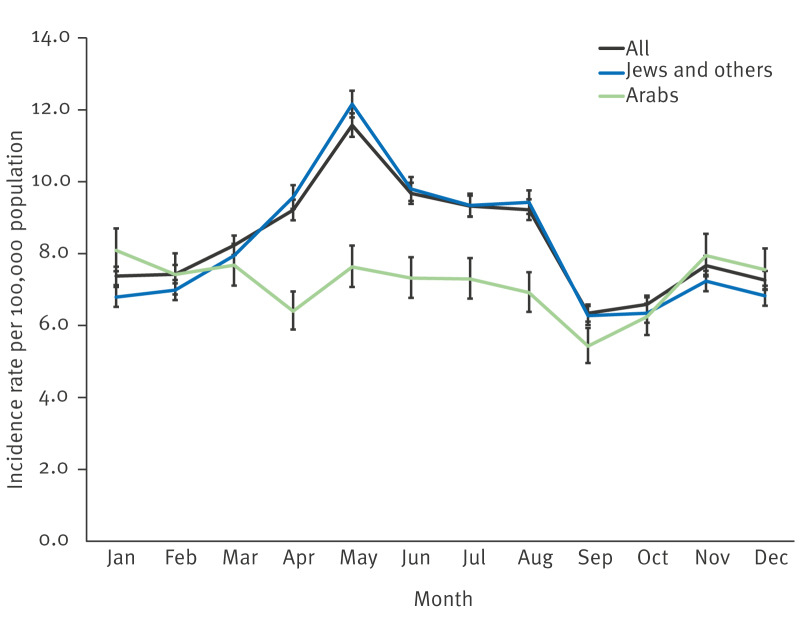
Mean incidence rate of campylobacteriosis, by month and population group, Israel, 2013–2022 (n = 43,334 cases)

Remarkably, the consistent annual April–May peaks occurred among Jews and others but not among Arabs (Figure 3). Incidence rate peaks during the study period by population group are presented in Supplementary Figure S5.

**Figure 4:**
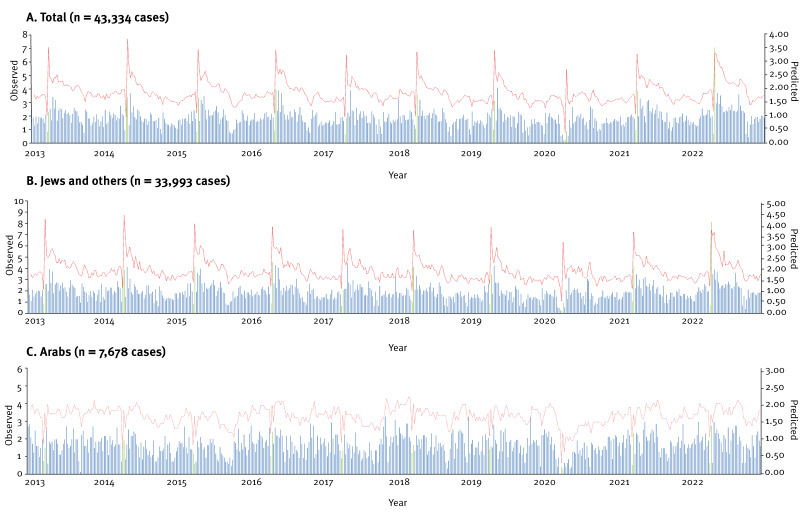
Observed and expected weekly incidence rate of campylobacteriosis per 100,000 population, in total, among Jews and others and Arabs, Israel, 2013–2022 (n = 43,334 cases)

Figure 4 shows the time series analysis and compares the observed and expected weekly incidence rate of campylobacteriosis in the total ISLBSN population, among Jews and others, and among Arabs. The models demonstrate a satisfactory alignment over Passover in the population group of Jews and others between the observed and predicted campylobacteriosis incidence rates. Passover as an explanatory variable for Jews and others accounted for 41.9% of the variance, and its adequacy was validated through the Ljung-Box test with a p = 0.775, while for Arabs, 18.4% and p = 0.844, respectively.

The blue bars (observed) refer to the notified incidence rate of campylobacteriosis, the red line to the predicted incidence rate of campylobacteriosis and the green bars represent the weeks in which Passover occurs.

Using Poisson regression, we estimated IRR and 95% CI for the risk of campylobacteriosis by week type (Passover vs non-Passover), age group, sex and population group, as presented in Supplementary Table S3. Passover weeks were associated with a significantly higher risk of campylobacteriosis (IRR = 1.18; 95% CI: 1.12 to 1.23; p < 0.0001) compared with non-Passover weeks.

During the study period, 12,155 (28.0%) of the *Campylobacter* isolates identified at the sentinel laboratories were species identified at the *Campylobacter* National Reference Center. Among these, 9,577 (78.79%) were identified as *C. jejuni*, 2,515 (20.69%) as *C. coli*, and 0.52% as other or non-identified species (50 (0.41%) *C.* spp., 8 (0.07%) *C. fetus*, 3 (0.02%) *C. lari* and 2 (0.02%) *C. upsaliensis*).

## Discussion

The estimated age-adjusted annual incidence rate between 2013 and 2022 was 83.0 per 100,000 population, notably higher than in the US (17.8/100,000) and the European Union/European Economic Area (EU/EEA) countries (44.5/100,000) in 2021 [14,15]. In a global study, Czechia had the highest incidence rate (215.0/100,000), followed by Australia (146.8/100,000) and New Zealand (126.1/100,000), while Bulgaria (3.3/100,000), Cyprus (2.4/100,000) and Poland (1.9/100,000) reported lower rates among the countries included [16]. These variations may reflect real differences in the incidence rates between countries, but also variations in laboratory detection methodologies and in surveillance and reporting systems.

We may have underestimated the incidence rates of campylobacteriosis as the data included only laboratory-confirmed infections. For salmonellosis, for example, in the US, there were an estimated 38.6 non-detected cases of *Salmonella* infection per every culture-confirmed case [17], while in Israel, each culture-confirmed case of shigellosis was estimated to represent 25.2 non-detected cases in the community [18].

We observed a significant decrease in the estimated age-adjusted incidence rate of campylobacteriosis between 2013 (101.7/100,000) and 2020 (79.4/100,000) and an increase thereafter up to 2022 (109.5/100,000). Similar trends were also reported by European Centre for Disease Prevention and Control (ECDC) pointing out a decrease in the notified incidence rate of campylobacteriosis between 2017 (63.3/100,000) and 2019 (59.9/100,000), which further dropped significantly in 2020 (42.8/100,000), largely due to the COVID-19 pandemic, with a slight increase in 2021 [15]. The US FoodNet reported stable rates from 2016 to 2021 [14]. The decrease observed in the incidence rate of campylobacteriosis in 2020 during the COVID-19 pandemic was consistent with other reports [14,15]. As previously reported by us, the relative risk reduction (RRR) for campylobacteriosis was 30.0% in March–July 2020 compared with the same period in 2018–2019, which was lower than the RRR reported for shigellosis (86.6%) and for salmonellosis (33.0%) [19]. Possible reasons for this could be reduced healthcare-seeking behaviour for mild diarrhoeal illnesses and decreased exposure to contaminated food due to fewer outdoor activities, which is often associated with outdoor gathering and barbecues. Most probably, the increased social distancing and hand washing during the early phase of the pandemic reduced person-to-person transmission of shigellosis, a disease highly endemic in Israel despite being a high-income country [20]. However, incidence rates increased in 2021 and 2022. This increase may reflect an actual rise in case numbers but also the introduction of detection by PCR, with higher sensitivity than the culture-based methods [10].

The estimated age-adjusted incidence rate of campylobacteriosis in Israel consistently displayed peaks in April–May. In the EU/EEA countries, a sharp increase of cases in the summer months (June–September) has been observed [15,21]. Interestingly, the consistent May peaks of campylobacteriosis were observed specifically in the population group of Jews and others, with no corresponding increase among Arabs. This suggests that cultural or nutritional factors may explain the differences. Differences in poultry consumption patterns between population groups may partly explain the observed disparities, as a previous study demonstrated that Jews and Arabs living in the same region in Israel exhibit major differences in dietary patterns, including lower reported poultry intake among Arabs [22]. The substantial rise in chicken meat and egg consumption in the Jewish population during the April Passover holiday could be associated with the increased incidence rate rather than climatic reasons. Previous studies in Israel have demonstrated an association between the frequency of chicken meat consumption and campylobacteriosis [23]. Indeed, in 2021, the demand for chicken in retail chains surged by 23% ahead of Passover compared with average sales in other periods [24], ranging between 21% and 46% in Israel from 2019 to 2023. The highest incidence rates of campylobacteriosis among Jews and others were recorded during Passover with no such rise in incidence rate among Arabs. High incidence rates during holidays have also been seen in other studies [25,26]. In 14 European countries, a winter peak in notified cases of campylobacteriosis has been seen, predominantly in calendar weeks 52 and 1, pointing towards risk exposures around Christmas and New Year [25]. Meat consumption habits and increased travel activities over the festive season may have been the contributors [25,26].

Like other studies, the incidence rate of campylobacteriosis in our study was highest among children aged 0-4 years [15,27]. The reasons for the higher incidence rates may be linked to transmission: first exposure to contaminated food and water, direct contact with animals, the faecal-oral route and higher likelihood to be referred by a paediatrician to perform a stool test. Acquired immunity against *Campylobacter* after repeated natural exposures could contribute to fewer cases in the adult population [28].

In the first year of life, the incidence rate among Arabs was 5.6 times higher than among Jews and others. This ratio remained high (1.2-fold) in favour of Arabs in the second year of life and inverted since the third year of life and presented higher risk in Jews and others compared with Arabs. A possible explanation is that the Israeli Arabs live predominantly in rural settlements [29], and Arab young children may have more direct contact with chickens and other animals.

The incidence rate among males was higher throughout the study period, as has also been reported previously by others [1,15]. Green et al. suggested that the male predominance in campylobacteriosis in early childhood is due, at least in part, to physiological or genetic differences and not behavioural factors [30].

*Campylobacter jejuni* was the predominant species (78.8%) identified, consistent with previous results from the ISLBSN [7], but at a lower proportion than that reported by the Centers for Disease Control and Prevention (CDC) and ECDC (ca 90%) [1,15]. This difference may reflect differences in sources of the infection or detection methods.

We may have underestimated the real incidence rate of campylobacteriosis since the analysed surveillance data included only laboratory-proven infections. There might be differences in the utilisation of medical services and health-seeking behaviour between the Jewish and Arab population groups, possibly leading to an underestimation of the incidence rate in Israeli Arabs, especially in older age groups. Although the sentinel laboratories cover only 50.5% of the Israeli population, their demographic composition closely reflected the national population by age group, sex and population group. Another limitation of this study is the variation in laboratory methods used for *Campylobacter* detection across sites and over time. While most laboratories transitioned from culture-based methods to PCR between 2020 and 2022, others continued using culture throughout the study period. Differences in the sensitivity and specificity between these methods, and across laboratories, may have introduced variability in detection rates. However, due to the lack of standardised performance metrics across all participating laboratories, it is difficult to fully quantify the extent or direction of this potential bias. In addition, the use of descriptive statistics may not fully account for potential confounding factors such as healthcare access, or diagnostic practices, which may influence observed incidence rates. We acknowledge that a multivariate Poisson regression model could offer additional insights. However, due to limited case counts in certain strata, such analyses were not feasible in the current study.

The rising antimicrobial resistance (AMR) in *Campylobacter* infections poses a serious public health challenge [31], complicating treatment and increasing the potential for severe outcomes. Unfortunately, our study did not include an analysis of the AMR patterns of the *Campylobacter* isolates with specific genetic or phenotypic characterisation.

The strength of our study relies on the usage of consistent and long-term systematic data collection.

## Conclusion

We observed a significant decline in incidence rates from 2013 to 2020, followed by an increase, consistent with global trends, though Israel's rates were higher. Children under 5 years consistently had the highest incidence rates, reflecting their greater vulnerability. We have shown that the morbidity peaks in April–May, largely driven by the Jewish population were associated with Passover weeks. To prevent future peaks of *Campylobacter* infections, particularly during holidays, it is essential to raise public awareness by publishing timely recommendations for consumers.

## Data Availability

Aggregated data can be made available upon request.

## Supplementary Data

417000 Supplementary Material
