# Supplementary material for: Trends in incidence and epidemiological characteristics of campylobacteriosis, Israel, 2013 to 2022
Source: Euro Surveill. 2025 Dec 4;30(48):2500181. doi: 10.2807/1560-7917.ES.2025.30.48.2500181 (PMC12680916; doi:10.2807/1560-7917.ES.2025.30.48.2500181)

This supplementary material is hosted by Eurosurveillance as supporting information alongside the article "Trends in incidence and epidemiological characteristics of campylobacteriosis, Israel, 2013 to 2022" on behalf of the authors, who remain responsible for the accuracy and appropriateness of the content. The same standards for ethics, copyright, attributions and permissions as for the article apply. Supplements are not edited by Eurosurveillance and the journal is not responsible for the maintenance of any links or email addresses provided therein.

Table S1. Comparison of demographic characteristics between the Israeli population and the sentinel laboratory population

Variable	Category	Israel population (%)	Sentinel laboratory population (%)
**Age group (years)**	0-4	10.3	10.7
	5-14	17.9	19.0
	15-19	7.8	8.2
	20-24	7.3	7.4
	25-29	7.0	6.7
	30-34	6.9	6.2
	35-44	12.8	12.5
	45-54	10.0	9.9
	55-64	9.0	8.6
	65-74	6.1	6.4
	75+	4.9	4.5
**Sex**	Male	49.6	49.6
	Female	50.4	50.4
**Population group**	Jews and Others	79.2	79.4
	Arabs	20.8	20.6

Table S2. Characteristics of patients identified with positive samples to *Campylobacter* in the ISLBSN (January 2013–December 2022)

Variable	Category	N	%
**Sentinel laboratory**	Haemek Medical Center	2,870	6.6
	Clalit Haifa HMO	9,873	22.8
	Maccabi Dan District HMO	3,553	8.2
	Soroka Medical Center	9,991	23.1
	Sheba Medical Center	1,271	2.9
	Hadassah Medical Centers	1,128	2.6
	Meuhedet HMO	8,642	19.9
	Clalit Petah-Tikva HMO	6,006	13.9
	Total	43,334	100.0
**Age group (years)**	0–4	17,897	41.5
	5–14	7,221	16.7
	15–19	2,867	6.6
	20-24	2,753	6.4
	25–34	3,550	8.2
	35–44	1,758	4.1
	45–54	1,376	3.2
	55–64	1,680	3.9
	65+	4,050	9.4
**Sex**	Male	23,796	55.0
	Female	19,443	45.0
**Birth Country**	Israel	37,495	87.8
	Other	5,208	12.2
**Population group**	Jews and Others	33,993	81.6
	Arabs	7,678	18.4
**District**	Jerusalem	7619	17.7
	North	7200	16.8
	Haifa	5613	13.1
	Central	5172	12.0
	Tel-Aviv	4780	11.1
	South	10032	23.3
	Judea and Samaria	2573	6.0
**Socioeconomic rank**	Median (IQR)	40,063	5 (3-7)

Table S3. Incidence Rate Ratios (IRRs) for campylobacteriosis based on Poisson regression analysis

**Variable**	**Category**	**IRR**	**95% Confidence Interval**	***p-value***
**Week type**	Passover vs. non-Passover	1.18	1.12-1.23	<0.0001
**Age group (years)**	0–4 vs. 55-64 years	8.10	7.71-8.52	<0.0001
	5–14 vs. 55-64 years	1.73	1.63-1.84	<0.0001
	15–19 vs. 55-64 years	1.82	1.72-1.94	<0.0001
	20-24 vs. 55-64 years	1.34	1.26-1.42	<0.0001
	25–34 vs. 55-64 years	0.73	0.68-0.78	<0.0001
	35–44 vs. 55-64 years	0.75	0.69-0.80	<0.0001
	45–54 vs. 55-64 years	1.83	1.73-1.93	<0.0001
	65+ vs. 55-64 years	1.79	1.69-1.89	<0.0001
**Sex**	Male vs. Female	1.25	1.22-1.27	<0.0001
**Population group**	Jews and others vs. Arabs	1.15	1.12-1.18	<0.0001

Figure S1: Geographic distribution of the laboratories included in the Israel Sentinel Laboratory-Based Surveillance Network

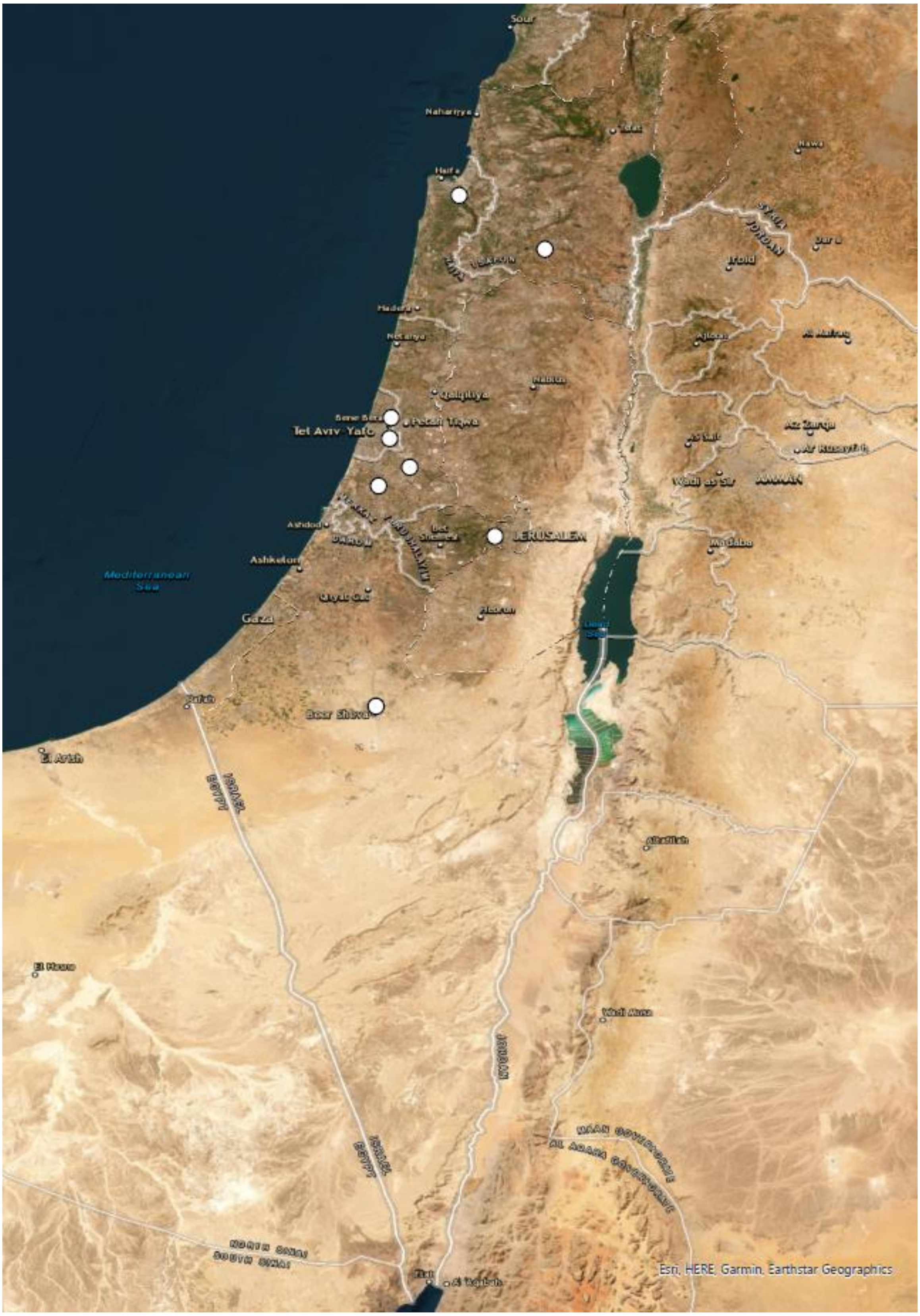

Figure S2. Incidence rates of campylobacteriosis per 100,000 by age group in Israel, January 2013–December 2022

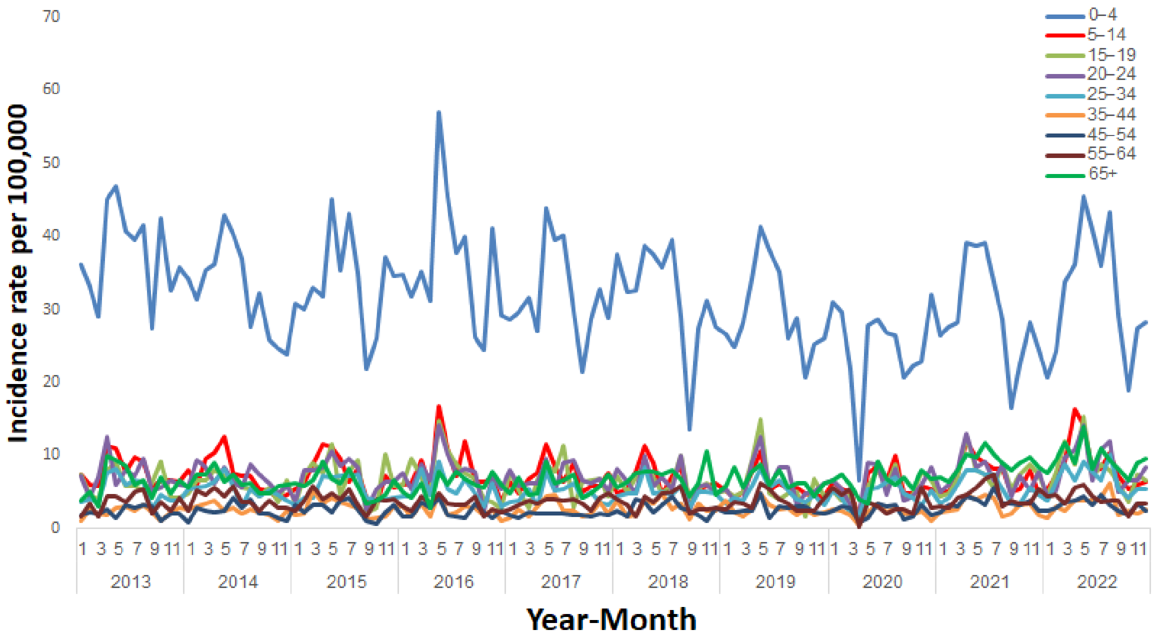

Figure S3. Incidence rates of campylobacteriosis per 100,000 by sex in Israel, January 2013–December 2022

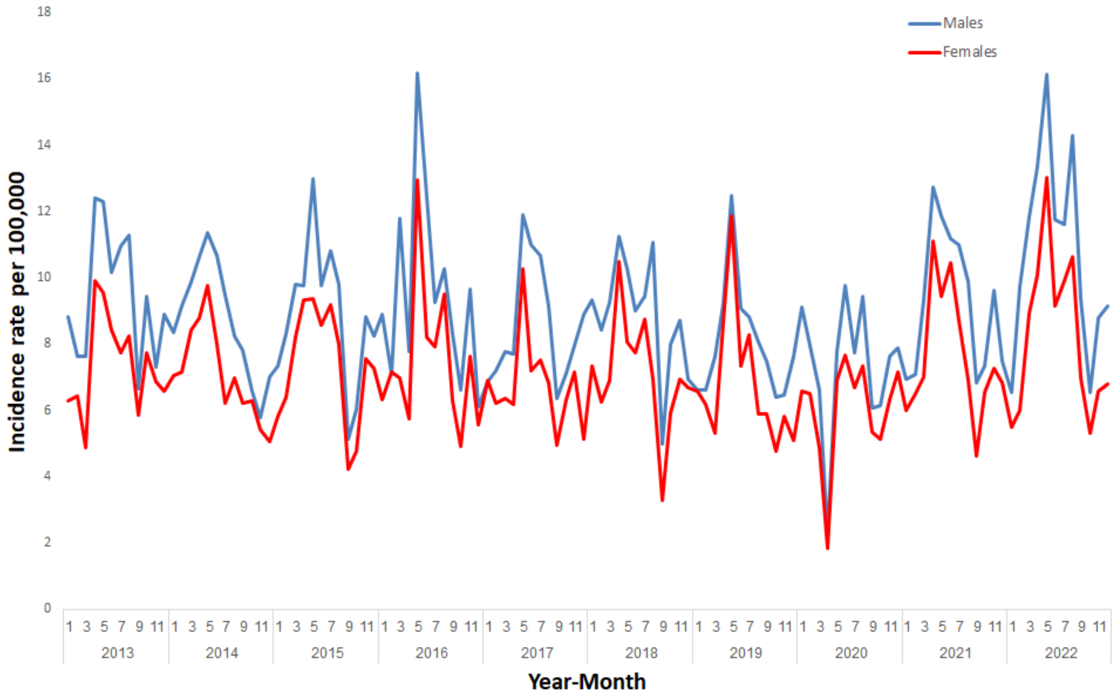

Figure S4. Mean incidence rate per 100,000 of campylobacteriosis by age group and population group in Israel, 2013–2022

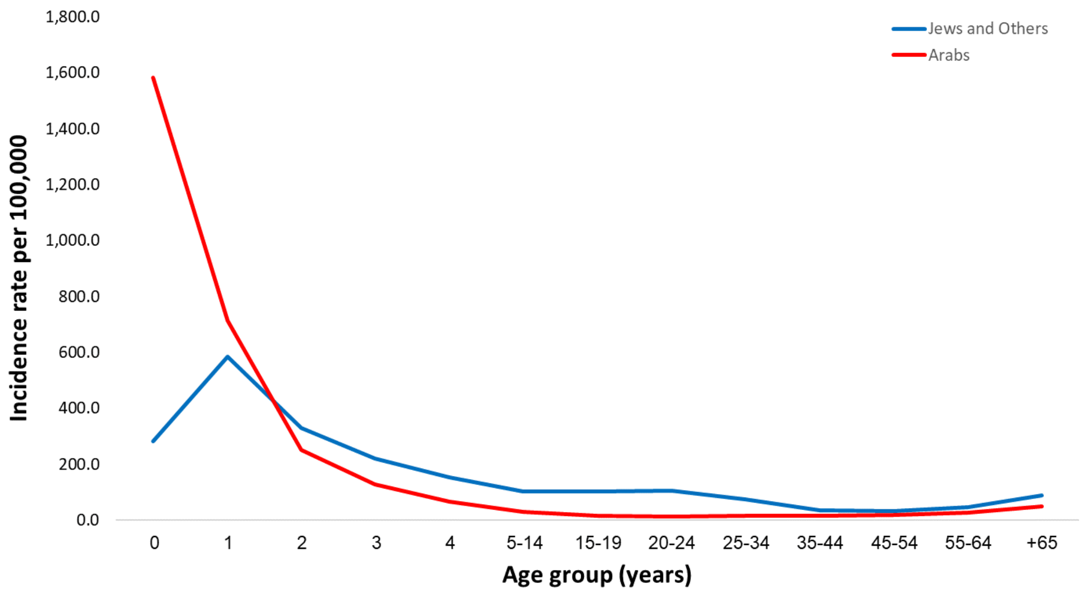

Figure S5. Observed incidence rates of campylobacteriosis per 100,000 by population group in Israel, January 2013–December 2022

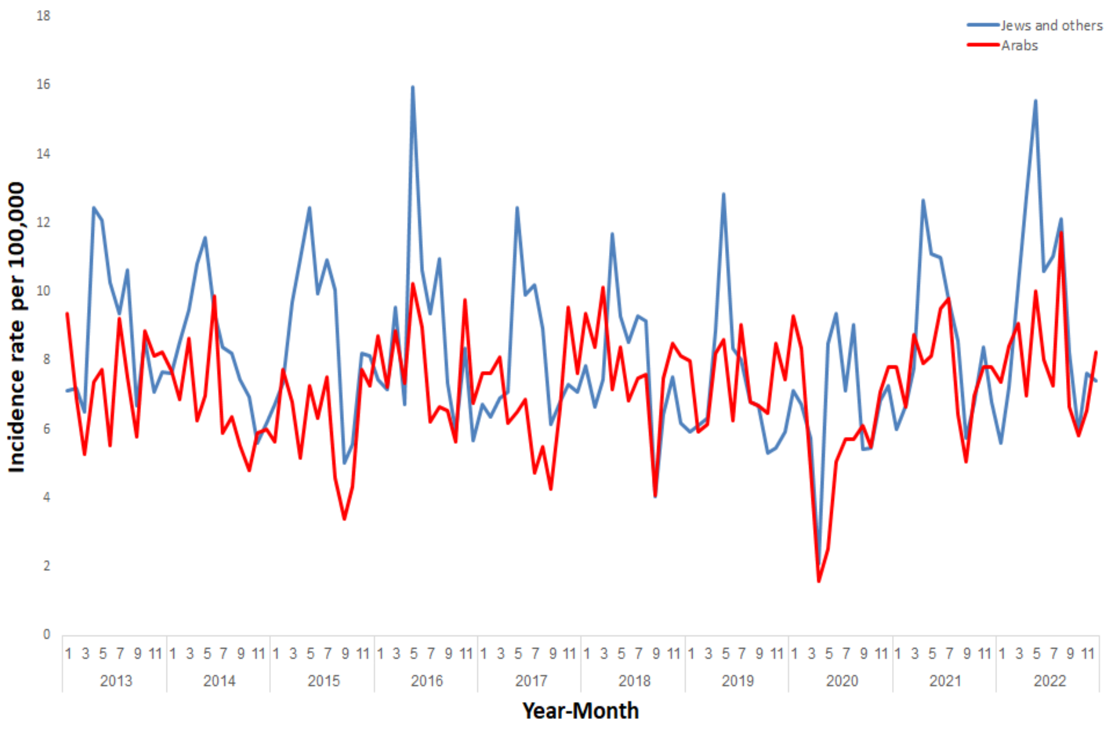

Supplement: Supplementary Material [file 25-00181_BASSAL_Supplement.pdf]
